# The CRISPR revolution in fungal biology and biotechnology, and beyond

**DOI:** 10.1186/s40694-018-0064-3

**Published:** 2018-12-20

**Authors:** Alexander Idnurm, Vera Meyer

**Affiliations:** 1 School of BioSciences, The University of Melbourne, Parkville Campus, Victoria, 3010 Australia; 2 Department of Applied and Molecular Microbiology, Institute of Biotechnology, Technische Universität Berlin, Gustav-Meyer-Allee 25, 13355 Berlin, Germany

As *Fungal Biology and Biotechnology* completes its fourth year, it is timely to reflect on how the journal is progressing and to consider how the research the journal publishes fits within the context of other developments in biology. The revolution in biology that has occurred since 2013, when the first discussions were initiated about the value of the journal that became *Fungal Biology and Biotechnology*, has been the meteoric rise of gene editing technologies by CRISPR–Cas9 or equivalent systems (Fig. 1). Thus, in this editorial we first provide an update on the journal, and then comment on the impact of gene editing on fungi and vice versa.

**Fig. 1:**
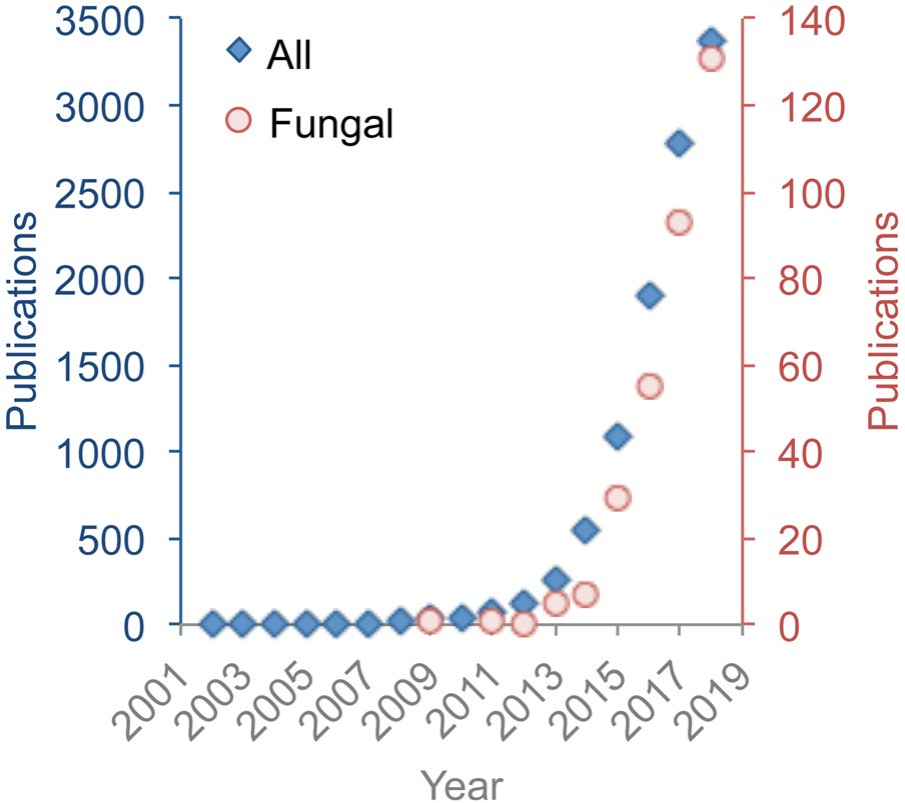
The use of CRISPR–Cas9 in fungal systems is on an exponential rise, and at the same pace with the rest of biology. The graph shows an annual increase in publication numbers for all papers in PubMed with CRISPR and with CRISPR + [fungus or fungi or yeast] as the search terms in the title or abstract. PubMed was accessed 2 November, 2018, and therefore the number of articles in 2018 will be higher

The last 12 months have seen yet again a strong performance from the journal, with its highest numbers of both submissions and publications to date. The original mission for the journal has continued as a guiding influence, with the journal supported by a vibrant editorial board [1] and through the actions of expert peer reviewers. The journal has also been supported with social media contributions in its twitter feed, particularly lead by Corrado Nai whose contributions in sharing the amazing world of fungal biology we acknowledge as he signs off from this role at the journal to undertake the next stage of his scientific adventure. Blog articles from junior investigators were developed after editorial board participation at the 14th European Conference on Fungal Genetics held in Haifa, Israel in February 2018. The journal had a 240% increase in the numbers of articles published from the previous time frame in 2016–2017, and as well a rise in citations in Google Scholar and Springer Citations to *Fungal Biology and Biotechnology* articles.

Changes in journals occur as a backdrop behind the driving impetus for publishing, which is by authors to share their new discoveries with the world. Meanwhile, a highly visible research change and one of the hottest topics during the last year has been the application and refinement of the CRISPR technology, including for fungi. The numbers of publications using this type of gene editing are growing at an exponential rate, with those featuring the fungi keeping pace (Fig. 1). The relationship of fungal research with gene editing takes two directions. In the first direction many fungi, and in particular those that are currently difficult to manipulate genetically, have and are likely to benefit from gene editing technology. In the second direction, fungi represent ideal hosts for dissecting how gene editing works or may be implemented for manipulating genes in eukaryotic cells.

The advancement that is driving the rate of adoption of CRISPR–Cas9 for many organisms is its capability of targeting a specific site in a genome: by and large trying to manipulate a piece of DNA using a homologous fragment of DNA is highly inefficient. One exception to this occurs in the yeast *Saccharomyces cerevisiae*, where gene replacements (or ‘editing’) can be achieved by transforming into cells fewer than 40 nucleotides of sequence divided on either side of a selectable marker. The high proportion of homologous targeting of exogenous DNA into the *S. cerevisiae* genome is one reason why yeast is such a powerful experimental tool. Indeed, relatively soon after its genome sequence was completed, every gene in the organism was deleted and those sets of mutants made available to researchers [2, 3]. Likewise, by using homologous integration all proteins were tagged with green fluorescent protein to understand subcellular protein localizations [4] and epitope-tagged to facilitate the global analysis of protein complexes [5]. Thus, it might be suggested that CRISPR–Cas9 is merely bringing other organisms up to the experimental levels enjoyed by the yeast research community; however, this gene editing system is already considerably beyond just a tool for making genetic changes.

Controversy resolves around CRISPR gene editing due to definitions about what constitutes ‘genetic modification’. We have been eating or using genetically modified organisms (GMOs) for thousands of years, with those modifications introduced by classical plant and animal selection and breeding efforts or the more recent rise of using transgenes in organisms to alter their traits. Pharmaceutical products have been produced with recombinant DNA technology for many decades. However, because the end result of gene editing can be free of any foreign DNA, and, if generated using recombinant Cas9 protein and RNA, have been made without even the use of DNA, CRISPR challenges previous concepts of ‘genetically modified’. Regulatory agencies are clearly scrambling to keep up with the technology. The fungi have played a leading role in decision making, wherein the most commonly eaten mushroom, *Agaricus bisporus*, was manipulated by gene editing and defined as not being regulated as a genetically modified organism (GMO) in the United States [6]. However, this freedom has been countered by the July 2018 decision of the European Court of Justice that CRISPR–Cas9 modifications should receive the same levels of regulation as what might be considered ‘conventional’ GMOs [7], drawing criticism from a number of researchers [8].

Given the role of CRISPR in driving new research, it is no surprise that research published in *Fungal Biology and Biotechnology* has featured this technique. For example, the journal published one of the first applications of CRISPR to the filamentous fungi, in the model species *Neurospora crassa* [9]. Other studies have reported on the use of this method for organisms in which it is almost impossible to make targeted mutations, such as *Leptosphaeria maculans* [10], the expression systems needed to synthesize the RNA to target the Cas9 endonuclease onto DNA, in *Aspergillus niger* [11], or to estimate how rarely the Cas9 endonuclease causes other changes in the genome, as in *Aspergillus fumigatus* [12]. Hence, these are four different stories, on four different fungal species, and with four different reasons to investigate their gene functions, illustrating both the wide appeal of the technique as well as the breadth of coverage of *Fungal Biology and Biotechnology.*

There is still much to learn about the potential unintended impact of using CRISPR–Cas9 in organisms, and more research is likely needed on the other genomic effects of the CRISPR method in fungi. How much ‘off-target’ mutation occurs is unclear, although this can and is already being assessed by re-sequencing the genome of an organism after gene editing has occurred. There is also a potential risk of larger scale chromosomal rearrangements [13]. One solution in a research laboratory setting is to complement any mutation that is created with the method, although in cases this requires some level of creativity to protect the new copy from also being targeted by the endonuclease. In a biotechnology scenario a wise course of action would be to ensure the removal of the gene editing machinery from any final product or to use an in vitro pre-assembled CRISPR–Cas9 machinery (ribonucleoprotein particles consisting of purified Cas9 protein and in vitro transcribed single guide RNA) for transformation, as shown for *Penicillium chrysogenum* [14].

While fungal species will benefit enormously from the availability of gene editing, the fungi continue to lead in our understanding about this new gene manipulation system well beyond within the fungi themselves, particularly through experiments in the model eukaryote *Saccharomyces cerevisiae*. This yeast was the first eukaryote organism manipulated with CRISPR–Cas9 [15] reported within months after the first publications on editing in mammalian cell lines [16, 17]. For instance, there is considerable variability in the efficiency of Cas9 targeting to different regions of the genome, recently shown in *S. cerevisiae* to be related to the chromatin context of the DNA to which the guide RNA targets [18]. In terms of applications, one exciting direction is the concept of using CRISPR–Cas9 to alter entire populations via an action as a gene drive, shown experimentally again in *S. cerevisiae* [19]. For filamentous fungi, this tool offers an excellent approach to generate minimal genomes for industrial cell factories, e.g. devoid of any secondary metabolite gene clusters encoding mycotoxins [20, 21]. Faster approaches to generate genome-wide mutant libraries are now possible, and this should lead to more examples of whole genome level gene manipulations in model fungi as was achieved in the pre-CRISPR era for *S. cerevisiae*.

One goal of *Fungal Biology and Biotechnology* is to promote fungal biology beyond the academic research sphere, and it is inspiring to see that CRISPR gene editing is being taught in university practical courses, using *S. cerevisiae* as the organism of choice [22]. CRISPR, using bacterial examples, is even being taught to children [23] and available for purchase as a do-it-yourself kit [24]. We thus commend the implementation of this technology into University lecture and practical courses, or at other education levels, to discuss together with the students the pros and cons of this technology for society, as well as legal and ethical aspects questions it may raise. We need to ensure that the new generation of students and scientists is informed about the application potentials and limitations of this technology and also be capable of discussing them with society. We can look forward to the contribution that *Fungal Biology and Biotechnology* will continue to make to the process of communication about new gene editing discoveries.

## Abbreviations

CRISPR: clustered regularly interspaced short palindromic repeats
Cas9: CRISPR associated protein 9
GMO: genetically modified organism
